# Contextual assembly of lexical functions in large language models

**DOI:** 10.3758/s13428-025-02898-7

**Published:** 2025-12-08

**Authors:** Christopher T. Kello, Polyphony Bruna, Kanly Thao

**Affiliations:** University of California, Merced, 5200 N. Lake Rd, Merced, CA 95076 USA

**Keywords:** Language models, Mental lexicon, Contextual assembly, Psycholinguistic ratings, Sensorimotor ratings

## Abstract

Neural network modeling has played a central role in psycholinguistic studies of lexical processing, but the recent advent of large language models (LLMs) offers a different approach that may yield new insights into the mental lexicon. Four LLMs were prompted across three experiments to test how they generate psycholinguistic ratings of words in comparison with humans. LLM ratings, averaged across varying list contexts, were found to be highly correlated with human ratings, and differences in correlation strengths were partly explained by differences in rating ambiguity. LLM context manipulations strengthened correlations with human ratings through better calibration, and variability in LLM ratings was correlated with human inter-rater variability. Additional results from testing LLM generation of word naming latencies showed functional deviations from factors that underlie human word naming, indicating that lexical function assembly in LLMs is currently limited by patterns of co-occurrence in textual data. Patterns at finer-grained timescales are needed in the training data to model online lexical processes. We conclude that LLMs used context to guide the assembly of generalized lexical functions, rather than recalling ratings and latencies from training data.

**Supplementary Information:**

The online version contains supplementary material available at 10.3758/s13428-025-02898-7.

## Introduction

The psycholinguistic properties of words have been studied for decades. Some properties reflect lexical processes of perception and memory, such as those engaged during word reading, and others reflect the usages and meanings of words in general and across various contexts. Models of lexical processing and semantics have played important roles in advancing our understanding of language, speech, reading, and cognition in general. Some of these advances have come from models of word embeddings that quantify how meanings can arise from context, and embeddings have become foundational to large language models (LLMs) as used in chatbots and other applications (Cassani et al., 2023).

As psycholinguistic models, word embeddings explain variability in how people use and process words (Mandera et al., 2017) and rate them along various psycholinguistic dimensions (Hollis et al., 2017; Mandera et al., 2015). Embedding models are based on the structures and similarities of learned representations in the form of normalized vectors. The representations serve as indirect proxies for psycholinguistic measures—for instance, correlation coefficients for state-of-the-art word embeddings with human ratings have been up to ~0.9 for variables like valence, arousal, dominance, age of acquisition, and concreteness (Vankrunkelsven et al., 2018).

We are interested in examining how LLMs may be used to model psycholinguistic and lexical processes more directly (e.g. Blank, 2023). For instance, generalized pre-trained transformer (GPT) models can be prompted as chatbots to generate text that can then be compared directly with human data, sometimes using the same prompts as given to human participants. In one study, several LLMs were tested in 12 psycholinguistic experiments, and the models replicated patterns of human language use in most cases (Cai et al., 2023). These patterns included assessing the meanings of unfamiliar words depending on their forms, determining the meanings of ambiguous words, syntactic priming, determining causality from verb semantics, and audience design. LLM language use also differed from that of humans in some ways, suggesting that LLMs may serve as models of some psycholinguistic processes but not others.

Recent studies have elicited ratings of words (Conde et al., 2025; Trott, 2024; Xu et al., 2025) and multi-word utterances (Martínez et al., 2024) from LLMs and compared them with human ratings. Word meanings can be assessed along psycholinguistic dimensions such as valence, concreteness, and arousal, as well as sensorimotor dimensions such as vision, hearing, and foot/leg actions. Trott (2024) collected GPT-4 ratings for 379 ambiguous words along nine psycholinguistic dimensions from the Glasgow norms (Scott et al., 2019) and for 112 words in different sentence contexts along 11 sensorimotor dimensions. Correlations between LLM and human ratings ranged from about 0.4 to 0.8. Martínez et al. (2024) collected GPT-4o ratings for thousands of multi-word utterances along the three dimensions listed above. Human ratings were from previous studies (Muraki et al., 2023; Warriner et al., 2013), and correlation coefficients were ~0.8 overall and ~0.9 for a validation set. Even coefficients up to 0.95 have been reported (Plisiecki & Sobieszek, 2024), but in that case, a transformer model was trained on a set of human ratings and then tested on extrapolation to untrained words.

The ratings studies reviewed above were exploratory or methodological in scope. They investigated the extent to which LLMs can be used in place of word embeddings or as tailored models to generate norming data that approximates human ratings without needing to collect new data from human participants (Aher et al., 2023; Dillion et al., 2023). The studies did not investigate how LLMs might yield insights into the human mental lexicon. Like their connectionist precursors (Christiansen & Chater, 2001), LLMs may be used to further our understanding of psycholinguistic processes as measured through language tasks. However, two issues need to be addressed.

One issue is that researchers are currently unable to train LLMs with the capabilities of advanced GPT and Gemini models. The advanced models appear best at simulating human behavior, so they may be most capable of modeling psycholinguistic processes. That said, model parameters and training data are proprietary and too large and complex to be amenable to direct analysis. Instead, researchers have applied experimental methods to investigate how LLMs work, analogous to human experimental methods (Binz & Schulz, 2023). Comparing human and LLM behaviors has become a new method of investigating language and cognitive processes (Mahowald et al., 2024), and we use this method herein.

The other issue is that LLM training data may include the norming studies in which human ratings and other measures are recorded. LLMs do not store training data in a random-access memory, but they can sometimes recall information verbatim (Bender et al., 2021; Carlini et al., 2021). So-called “data contamination” may affect tests of psycholinguistic processes in LLMs if their responses are recalled from published studies (Deng et al., 2023). Such recall would not serve as a model of psycholinguistic processes because participants do not respond to words based on stored knowledge of how other participants have responded.

While data contamination is possible, LLMs are also capable of generalization from training data (Wang et al., 2024), e.g., based on the superposition of learned vectors across connection and attention weights (Jiang et al., 2024). Mechanisms of LLM generalization may provide useful insights into human psycholinguistic processes. For instance, LLMs exhibit emergent phenomena (Wei et al., 2022) whereby they learn to perform tasks not explicitly targeted by their objective function, which is to predict upcoming tokens based on their contexts in training data. This emergent functionality is also known as *in-context learning* (Dong et al., 2022), and it bears some resemblance to how humans can assemble context-dependent functions and performances “on the fly” based on generalizations from prior experiences.

In the present study, we test the degree to which LLMs perform lexical tasks based on context-dependent generalizations versus recall of training data. We prompted four LLMs through their chat application programming interfaces (APIs) to generate responses to English words for which human data are available in the South Carolina Psycholinguistic (SCOPE) Metabase (Gao et al., 2023). SCOPE is a compilation of psycholinguistic properties of words measured across several major studies of word recognition, word naming, and semantics.

We replicate previous studies in testing correlations between mean human and LLM responses, and we add to this growing body of literature by testing:

1. New variables chosen to adjudicate between generalization versus recall mechanisms;
2. Differences in correlations across variables with respect to the degree of ambiguity in how variables are applied to words; and
3. Relationships between contextual variability in LLM ratings and human inter-rater variability.

Altogether, we find evidence that LLM responses to word stimuli are driven by generalizing patterns of word co-occurrences in language rather than recalling data from norming studies. We end with a discussion of how our results inform the use of LLMs for generating psycholinguistic data and how lexical processes in both LLMs and humans may be assembled in context based on distilled patterns in language and interaction with the world.

## Experiment 1: Psycholinguistic word ratings

LLMs extract patterns of word usage from large training corpora, so they are well suited for modeling the semantic properties of words, along with structural and conceptual relationships among them. Moreover, chat interfaces to LLMs allow researchers to prompt word ratings using the same or similar instructions given to human participants. Experiment 1 focused on ratings of words along a range of psycholinguistic properties from three different norming studies. We extend Trott's (2024) study by including variables from two additional studies and investigate LLMs' ability to account for both the central tendency and variability of human ratings. We also test whether differences in accounting for human ratings across variables can be explained by the ambiguity of psycholinguistic variables.

### Methods

The psycholinguistic properties investigated in Experiment 1 were primarily taken from the Glasgow norms (Scott et al., 2019) that contain ratings for 5500 English words on a scale of 1 to 7 for six psycholinguistic variables and a scale of 1 to 9 for three additional variables. The first group of variables is age of acquisition, concreteness, familiarity, gender association, imageability, and semantic size, and the second group is arousal, dominance, and valence. Two additional variables were chosen from two other rating norms studies: Humorousness ratings for nearly 5000 English words on a scale of 1 to 5 (Engelthaler & Hills, 2018) and socialness ratings for 8388 English words on a scale of 1 to 7 (Diveica et al., 2023). The SCOPE metabase was queried for all words that appeared in all three corpora and that also had data for the speeded response variables and associated word properties examined in Experiment 3. We wanted stimuli to be the same across experiments so we could compare results. The intersection of these constraints resulted in a total of 390 English words investigated in Experiments 1 and 3.

The means and standard deviations (SDs) of ratings were published for each word and each of the 11 psycholinguistic variables across the three corpora. The number *N* of participant ratings was in the range [23,70] per variable per word, with a mean of 33.5 for the Glasgow norms. For humorousness, *N* = [19, 51] with a mean of 33.3, and *N* = [17, 25] with a mean of 21.9 for socialness. The prompts that elicited human ratings for each variable were also published, and we used them to elicit ratings from LLMs, with some minor edits to allow for batch responses to lists of words (see Supplementary Materials).

Ratings in norming studies were collected by presenting one word and getting one response per trial, with each experimental session consisting of a randomized list of about 100 to 150 words. While words were presented in isolation, earlier words can affect later ratings through calibration and comparison in memory. To simulate the possible effects of list composition, we also prompted LLMs with randomized word lists so that their ratings would be generated in the context of other words.

Preliminary tests indicated that all models could respond reliably to lists of 50 to 100 words, so the 390 stimuli were divided into five lists of 78 words each. A given list was appended to each prompt and submitted through the API to generate ratings for all 78 words, i.e., zero-shot prompting. Prompts were edited to allow for decimal responses on a continuous rating scale so LLMs could recall precise values from their training data (the studies reported mean ratings out to multiple decimal places). For example: “Valence is a measure of value or worth. A word is NEGATIVE if it represents something considered bad, whereas a word is POSITIVE if it represents something considered good. Rate the valence of each of the following words on a continuous scale from 1.0 (VERY NEGATIVE) to 9.0 (VERY POSITIVE), with the midpoint representing NEUTRAL:”

Prompts were delivered and responses collected through the OpenAI and Gemini API interfaces in Python. In addition to the user prompt, APIs also include a system prompt that sets the background context and instructions. The system prompt for all variables and models in Experiment 1 was set to the following: “The user will describe a feature of words and how to quantify it and assign values to words. The user will then provide a list of words. Respond only with the list of words and their corresponding values, one 'word, value' pair per line.” This prompt guided LLMs to generate responses in a standardized format that could be analyzed directly, without needing to filter out preamble and extraneous comments that chat interfaces are sometimes prone to producing.

### Results

Results for three GPT models and one Gemini model are presented here: gpt-3.5-turbo-0613, gpt-4–0613, gpt-4o-2024-08-06, and gemini-2.0-preview. Some data were also collected for other recent proprietary models, but results were essentially the same—results for a highly rated open-source model were substantially less human-like, which supported our choice to focus on proprietary models (see Supplementary Materials). The temperature was set at its default of 1, and the context window size was set to 4096. All statistical analyses herein were performed in the R environment using the following packages: *Tidyverse* (Wickham et al., 2019), *lmerTest* (Kuznetsova et al., 2017), *ggpubr* (Kassambara, 2018), *ggh4x* (van den Brand, 2023), and *patchwork* (Pedersen, 2019).

For each model, ten sets of ratings were collected for all 390 words and all 11 psycholinguistic rating variables. For each set of ratings, words were placed randomly in five lists of 78 words each. Ratings could vary across runs due to stochasticity in the next-token (softmax) function, and they could also vary as a function of list composition. Intra-word means and SDs of LLM ratings were computed for each psycholinguistic variable and compared with the corresponding human means and SDs.

First off, LLM ratings never used the decimal precision that was reported in the norming studies. Despite prompts that invited LLMs to use decimal precision, responses mostly consisted of integer or half-integer (X.5) ratings. On a few occasions, rating precision was given in tenths, but the published means of human ratings were all more than one decimal of precision—thus, LLMs did not recall and respond with published means verbatim.

#### Rating means

**Fig. 1:**
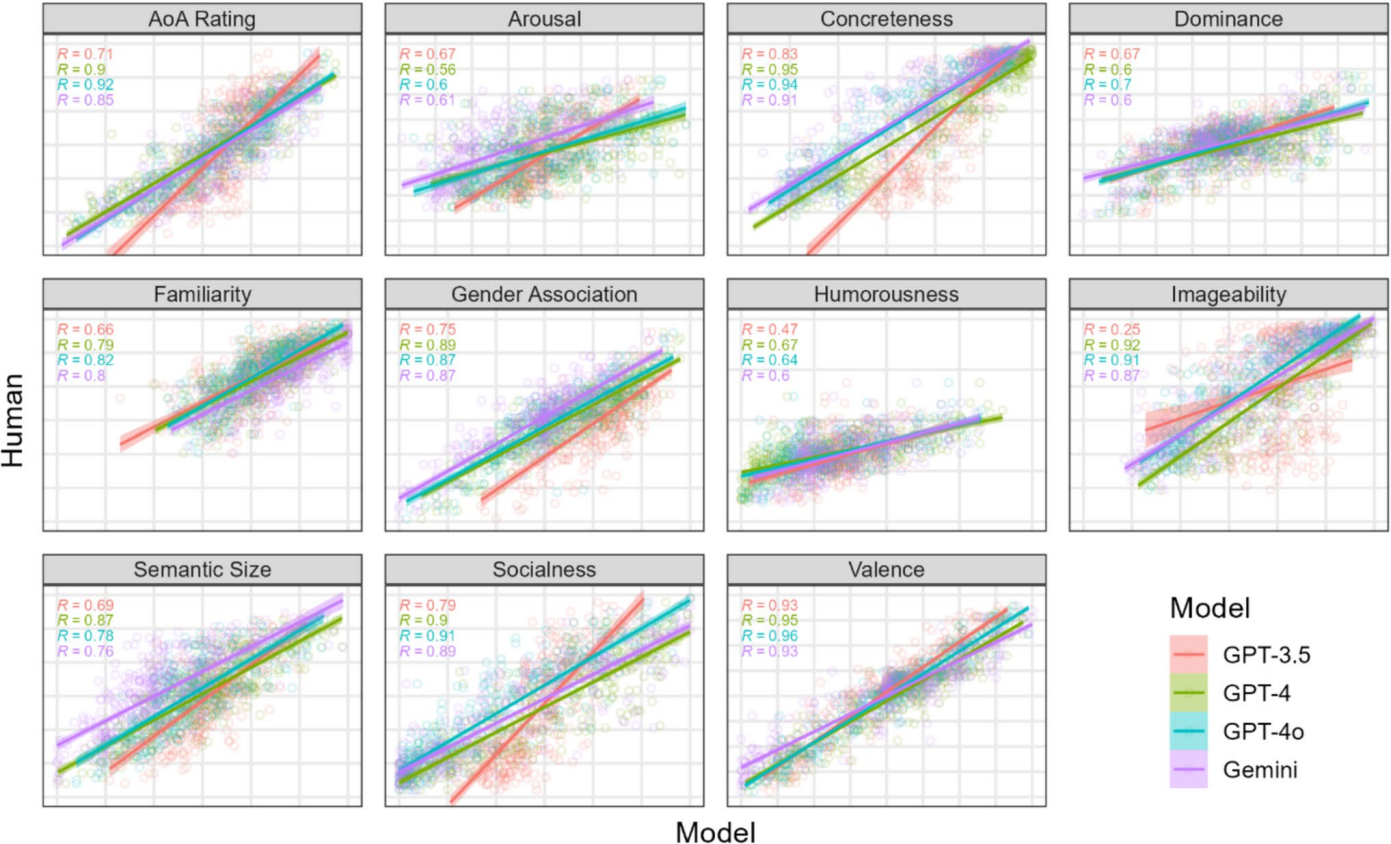
Scatter plots of LLM ratings (*X*-axis) against human ratings (*Y-axis*) for each psycholinguistic variable in Experiment 1, with 390 points per plot corresponding to the respective human and LLM rating means for 390 test words. The ranges of *X*- and *Y*-axes always matched and were set according to the scales of their respective variables (1 to 5, 7, or 9, as shown by grid lines). Multiple correlation coefficients *R* for each model are shown in the *upper left corne*r of each plot

Results for mean ratings are shown in Fig. 1, which contains 11 plots of LLM means against human means, one plot per variable. Each plot contains 390 points, one per word, for each of the four models. Linear trend lines are also plotted, along with correlation coefficients (R values) for each model and variable. Axes range from 1 to 5, 7, or 9, depending on the rating scale, which means that a perfect correspondence between human and LLM ratings would appear as all points falling along the diagonal of each plot.

Generally, there was a close correspondence between mean human and LLM ratings that were weaker for the older GPT-3.5 model. This apparent difference was tested using a mixed effects model with human rating means as the dependent variable, LLM rating means as the predictor variable, LLM model as a fixed effect, and word as a random effect. Human and LLM ratings were *Z*-scored with respect to each LLM model and psycholinguistic variable so that the mixed effects model tested differences in effect size between GPT-3.5 and the other three models: Human Rating ~ LLM Rating * LLM Model + (1 | Word). Results showed that human-LLM rating relationships were weaker for GPT-3.5 compared with GPT-4 (*b* = 0.14, *p* < 2e-16), GPT-4o (*b* = 0.14, *p* < 2e-16), and Gemini (*b* = 0.14, *p* < 2e-16).

Trott (2024) also tested GPT-4 on the nine Glasgow variables and found lower correlations with human ratings compared with our results, although correlations herein followed a similar pattern to those reported by Trott: We computed a Fisher *Z*-transform on our coefficients and on Trott’s, and we found them to be correlated with each other (*r* = 0.64). There are three possible reasons for higher correlations in our study:

1. We tested a different set of words;
2. We left temperature at its default setting of one which adds stochasticity to the LLM next-token output layer, whereas Trott set temperature to zero thereby making LLM responses mostly deterministic; and
3. We prompted each word ten times in ten different randomized lists of words and took the mean rating, whereas Trott prompted each word once in isolation through individual API calls.

We tested the role of stochasticity in our results by rerunning Experiment 1 with the same stimulus lists but with the temperature set to zero. This setting reduced intra-word variability by about 18–25% for GPT models compared with the default temperature (only 4% reduction for Gemini), but correlation coefficients for rating means were unaffected by the difference in temperature (< 0.01 difference across variables). We can conclude that variability in context, i.e., due to list composition, was the primary driver of intra-word variability that contributed to LLM rating means and their correlations shown in Fig. 1.

Figure 1 also suggests that the relationship between human and LLM ratings may be stronger for some psycholinguistic variables compared with others. Given that we did not have *a priori* hypotheses about the relative strengths of relationships across variables, we first ran an omnibus test for whether the strength of human–LLM relationships varied across the fixed effect of psycholinguistic variable, with word as a random effect. The omnibus test was highly reliable (*χ*2(10) = 660.22, *p* < 2.2e-16), which gave us license to investigate further human–LLM rating relationships across psycholinguistic variables.

We considered the three psycholinguistic variables with the weakest correspondences between human and LLM ratings: Arousal, dominance, and humor. Their prompts suggested that the meanings of these variables may have been ambiguous; the prompt for arousal did not mention its sexual connotations, the locus of dominance was left unspecified, and the prompt for humorousness included both genuine (e.g., hilarious) and cynical (e.g., laughable) connotations. If so, ambiguity may increase the variability of human ratings and thereby muddy the variability across words that is explainable by LLMs.

We tested this hypothesis by examining human–LLM correlations across psycholinguistic variables with respect to the average human SD of intra-word ratings, as a measure of inter-rater reliability. Our reasoning is that more ambiguity in applying a psycholinguistic variable to words, due to either conceptual vagueness or individual differences in usage, should result in less reliability and hence *larger* inter-rater variance. Consistent with the hypothesis, correlation coefficients were negatively correlated with average SDs across variables, *r* = – 0.32, *t* = – 2.35, *p* < 0.05.

#### Rating SDs

Next, we examined the relationship directly between human and LLM intra-word SDs, using the same correlational analyses as for human–LLM rating means. If there are underlying similarities in how human and LLM ratings are generated, then the ambiguity in applying a given psycholinguistic variable to a given word should be similarly reflected in human inter-rater variability, as well as LLM intra-word rating variability. As noted earlier, variability in list composition should contribute to both human and LLM variability.

**Fig. 2:**
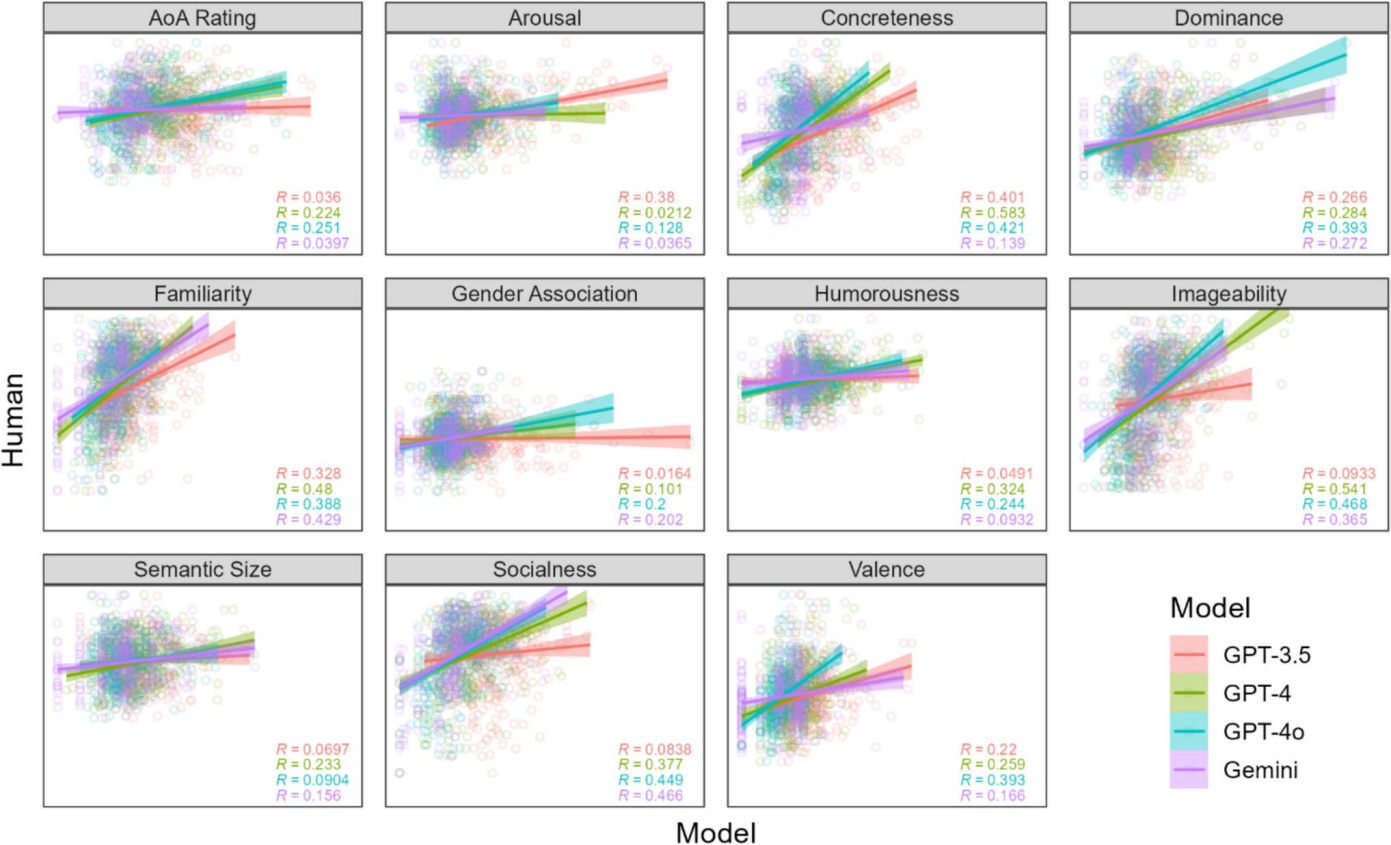
Scatter plots of LLM rating SDs (*X*-axis) against human rating SDs (*Y*-axis) for each psycholinguistic variable in Experiment 1, with 390 points per plot corresponding to the respective human and LLM rating SDs for 390 test words. Multiple correlation coefficients (*R*) for each model are shown in the *bottom right corner* of each plot. *X-* and *Y*-axis ranges are the same within each plot and set to minima and maxima of each variable, so a perfect positive relationship falls along the diagonal

Results for intra-word SDs are shown in Fig. 2, which is formatted the same as Fig. 1. Plots show a positive relationship overall in the SDs of human and LLM rating, although the relationship is weaker than for means and differs more across models and psycholinguistic variables. Correlation coefficients ranged from about 0.0 to 0.5, and those for GPT-3.5 were again weaker on average compared to the other models. Using a linear mixed-effects model with LLM rating SDs as a predictor of human SDs (both *Z*-scored) and a random effect of word, we found that LLM rating SDs were reliable predictors of human SDs overall (*b* = 0.20, *p* < 2e-16). This relationship was weaker for GPT-3.5 compared with GPT-4 (*b* = 0.14, *p* = 6.01e-13), GPT-4o (*b* = 0.12, *p* = 1.36e-10), and Gemini (*b* = 0.05, *p* = 0.018).

As with rating means, we again found that the strength of the relationship between human and LLM rating SDs differed reliably across psycholinguistic variables, using the same omnibus test as for rating means (*χ*2(10) = 205.88, *p* < 2.2e-16). To account for these differences, we reasoned that variables with more highly correlated SDs should be those whose interpretation is more contextual, in which case intra-word variability in LLM ratings should better reflect the degree to which words are affected by context. More contextual variables should have less variance in ratings across words when taken out of context by virtue of reverting to some default rating. Consistent with this hypothesis, the human–LLM correlation of rating SDs increased as the SD of rating means decreased, *r* = – 0.22, albeit unreliably: *t* = – 1.5, *p* < 0.15.

### Experiment 1 summary

Correlations between human and LLM mean ratings were all highly reliable and stronger on average compared with correlations reported in previous studies, with about half of the coefficients above 0.9 for the more advanced models. Such human-like LLM ratings may result from the way that LLMs generalize patterns of word co-occurrences, or they might reflect data contamination, i.e., recall of published ratings from norming studies that were included in LLM training data.

The recall account is challenged by three results. First, individual LLM ratings did not match the published rating means, as one would expect if LLMs recalled means from their training data. Instead, the average of ten ratings per word converged on the human rating means. Data contamination might account for this finding if training data included individual human ratings rather than just means and standard deviations, but only the latter were published in the Glasgow norms. Nevertheless, it is possible that multiple ratings per word and per variable are in the training data for LLMs to average in their responses, but this recall account runs into a second challenge: Why were LLM ratings less correlated with human ratings for more ambiguous variables? Generalization can explain the differences based on the degree of ambiguity in how different variables are applied to different words. A recall account would need to posit fewer available ratings to average for more ambiguous variables, but we see no rationale for such a claim.

The third challenge for the recall account is the correspondence between human and LLM rating *variability*. LLMs cannot use recalled rating SDs to control the variability of their responses across independent API calls. If training data include more dispersed ratings for words with greater SDs, one might imagine there is some way that this data contamination could cause LLM responses to be more variable. We know of no evidence for such a mechanism, and as noted above, the Glasgow study did not publish individual ratings. Thus, the correspondence between human and LLM rating variability casts further doubt on the recall account, whereas this finding is readily explained by the generalization account: Variability in word lists caused variability in assembled ratings functions, and this variability was influenced by the diversity of word usage in training data. We continue to test these competing accounts using a different approach in the following experiment.

## Experiment 2: Context effects

Context effects in human lexical processing are widespread (Lucas, 1999), including the effects of the composition of word lists (Dorfman & Glanzer, 1988). These effects likely reflect the inherent contextuality in how words are interpreted in natural language use based on a range of linguistic and metalinguistic factors (Pustejovsky & Boguraev, 1995). The generalization account explains results from Experiment 1 in terms of similarities in how humans and LLMs assemble lexical functions based on generalizations from experience for humans and from training data for LLMs.

In Experiment 2, we tested the effect of context on LLM word ratings using the Lancaster sensorimotor norms (Lynott et al., 2020). In this study, participants were asked to rate words according to their degree of involvement for six perceptual modalities (touch, hearing, smell, taste, vision, and interoception) and five action effectors (mouth/throat, hand/arm, foot/leg, head excluding mouth/throat, and torso). The norms were designed to tap into sensorimotor experiences associated with words, but LLMs lack such experiences. Nevertheless, patterns in word co-occurrences may provide a basis for LLMs to infer the degrees to which different modalities and effectors are associated with different words (Banks et al., 2021).

The Lancaster corpus includes the specific lists of words that participants saw. We presented LLMs either with original word lists or the same words but rearranged into differently composed lists. We gathered LLM ratings in both conditions and compared them with human rating means and SDs, as in Experiment 1. If LLMs assemble lexical functions based on generalizations from training data conditioned on context, then the correspondence between human and LLM ratings should vary as a function of list composition. By contrast, the recall account has no ready basis for predicting systematic effects of list composition on the retrieval of published means and SDs from training data.

### Methods

Perception modalities and action effectors were tested separately in the Lancaster study. Each test consisted of a list of 58 words and two-word phrases to be rated on a scale of 0 to 5, where 0 meant that the word is not at all involved with the modality or effector, and 5 meant it is greatly involved. Participants rated each word on all six perceptual modalities or all five action effectors, and the left-to-right order of modalities or effectors to be rated was randomized anew for each list presentation. Each stimulus list started with five calibrator words followed by five control words mixed with 48 test words in a newly randomized order per presentation. Ten calibrator and control words were created to be used for all perception lists, and ten different calibrator and control words were created to be used for all action lists.

Eight perception lists and eight action lists (data for about 19 participants on average per list) were chosen at random to be used for Experiment 2, resulting in 384 words for testing perceptual modalities and 384 different words for testing action effectors (about the same as the 390 words used in Experiment 1). Calibrator and control words were also included, as in the original study, but were not analyzed as test words. The action and perception user prompts were copied from prompts in the Lancaster study and edited to guide the formatting of chatbot responses along with the system prompt (see Supplementary Materials). As in the Lancaster study, the order of modalities was randomized each time.

There were two list composition conditions. In the *original* condition, each of the original 16 lists was presented to each of the four LLM models tested in Experiment 1. To isolate and observe any possible effect of context, we wanted a comparison condition that was maximally different from the original lists, which were randomized. In the binned condition, we created lists that were concentrated on ratings in one modality instead of being evenly distributed. Specifically: (1) The set of 384 words was sorted according to Mouth or Vision ratings; (2) each sorted word set was divided into eight lists of 48 words each; (3) five Mouth or Vision exemplar words (see below) were added to each respective list; (4) each list was randomized and five Mouth or Vision calibrator words were appended to the start.

We chose the sorted modality and effector to be those with the largest number of words with maximal ratings in the entire Lancaster corpus, which were the Vision modality and the Mouth effector. We then chose calibrator and control words to be the ten words with maximal Vision ratings in the whole corpus, and ten words with maximal Mouth ratings. Thus, the binned lists focused on bands of Vision and Mouth ratings, and calibrator and control words exemplified maximal Vision and Mouth ratings. Zero-shot prompts were used as in Experiment 1, which means that the LLMs were not given ratings to calibrate; they were only given the words, whose presence in word lists served as context that might help LLMs self-calibrate.

If LLMs recall ratings from the Lancaster study, one might expect recall to be facilitated when the original word lists are used. The study did not include those lists directly, so one might also expect no effect of list composition. By contrast, if the context of list composition helps to calibrate the assembly of lexical functions, then the binned condition might lead to better calibration and better correspondence with human ratings for the Vision and Mouth variables. One might also argue the opposite effect, given that the binned lists provide a different context compared with the original study.

### Results

As in Experiment 1, each list was presented ten times with ten new randomizations (calibrator words were always the first five) to each of the four LLM models. LLM rating means and SDs were computed for each word or phrase, and each LLM model, in both the original and binned context conditions.

#### Rating means

**Fig. 3:**
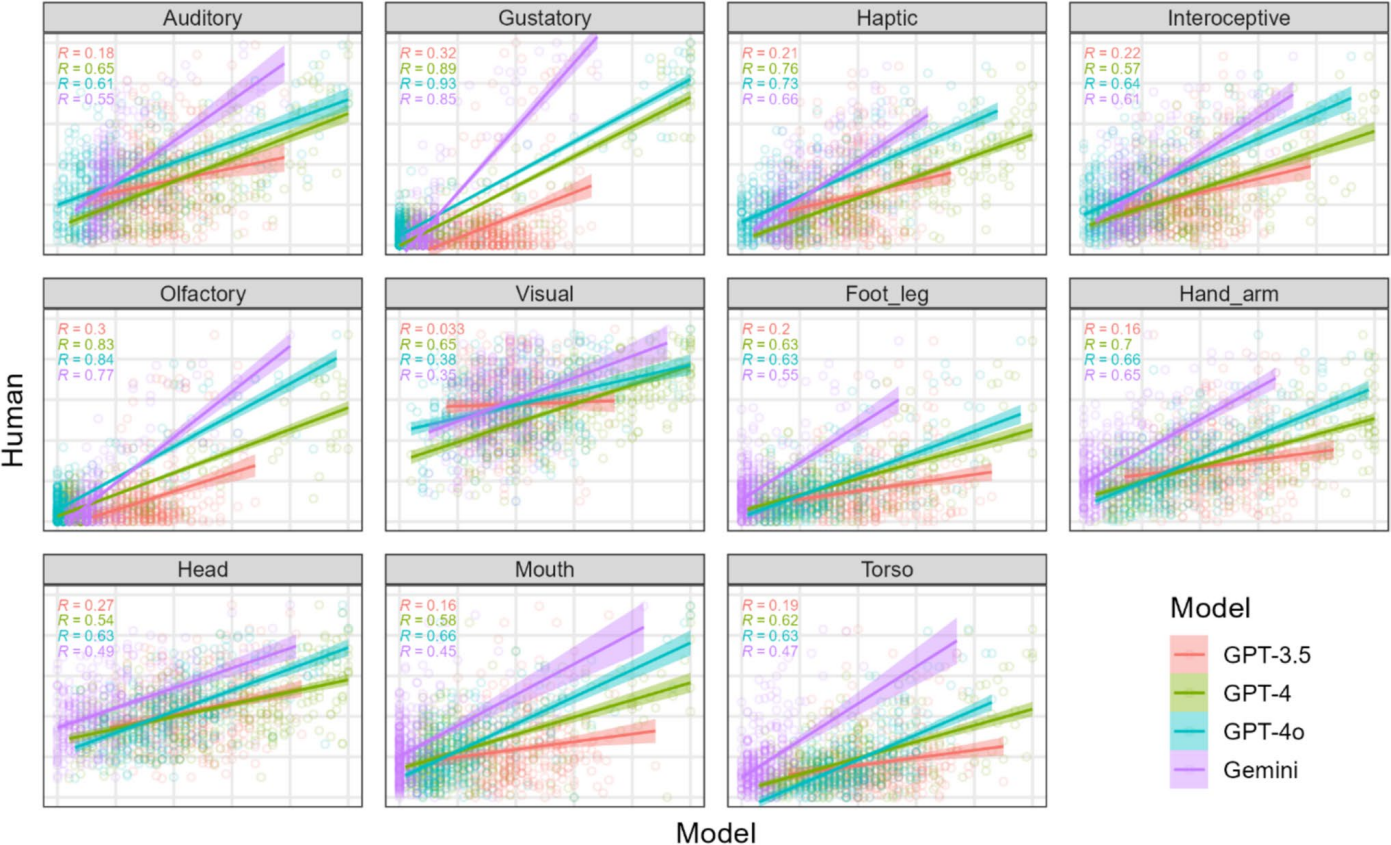
Scatter plots of LLM ratings (*X*-axis) against human ratings (*Y*-axis) for each sensorimotor variable in the original condition of Experiment 2, with 385 points per plot corresponding to the respective human and LLM rating means for 385 test words. *X-* and *Y*-axes ranged from the minimum to maximum rating (0 to 5) in all plots. Multiple correlation coefficients *R* for each model are shown in the *upper left corner* of each plot. A perfect correspondence between human and LLM mean ratings would appear as all points falling along the diagonal of each plot

Correspondences between human and LLM rating means are shown in Fig. 3 for the original lists. The positive trend lines and *R* values show moderately strong relationships for the Lancaster norms, albeit weaker on average than those found for the psycholinguistic variables in Experiment 1. LLM rating responses were integers and prompts did not invite decimal values.

As in Experiment 1, the more recent and advanced LLMs produced word ratings whose means more closely followed that of human raters. The mixed effects model with *Z*-scores from Experiment 1 was used with words, mean ratings, and variables from Experiment 2. The model showed again that GPT-3.5 rating means were less correlated with human rating means compared with more advanced models: *b* = 0.49, *p* < 2e-16 for GPT-4; *b* = 0.48, *p* < 2e-16 for GPT-4o, and *b* = 0.42, *p* < 2e-16 for Gemini.

Figure 3 also suggests that the correspondence between human and LLM rating means may be different across sensorimotor variables. The omnibus test used in Experiment 1 was adapted for the 11 sensorimotor variables and a reliable difference was found: χ^2^(10) = 243.95, *p* < 2.2e-16. Overall, LLM ratings were more strongly correlated for perceptual modalities than motor effectors (*b* = 0.10, *p* = 9.46e-15) and for gustatory compared to most other perceptual variables: *b* = – 0.18, *p* = 4.88e-10 for auditory; *b* = – 0.10, *p* = 0.000316 for haptic; *b* = – 0.18, *p* = 1.37e-10 for interoceptive; *b* = – 0.05, *p* = 0.079669 for olfactory; *b* = – 0.33, *p* < 2e-16 for visual.

Like Experiment 1, we sought to explain some of the variance across sensorimotor variables in terms of dispersion of ratings across words. Again, we examined human-LLM correlations across variables with respect to the average human SD of intra-word ratings, as a measure of inter-rater reliability. Correlation coefficients were again negatively correlated with average SDs across variables, *r* = – 0.36, *t* = – 6.25, *p* < 0.00001, consistent with the idea that ambiguity in applying a variable reduces the ability of LLMs to model its mean ratings.

#### Rating SDs

**Fig. 4:**
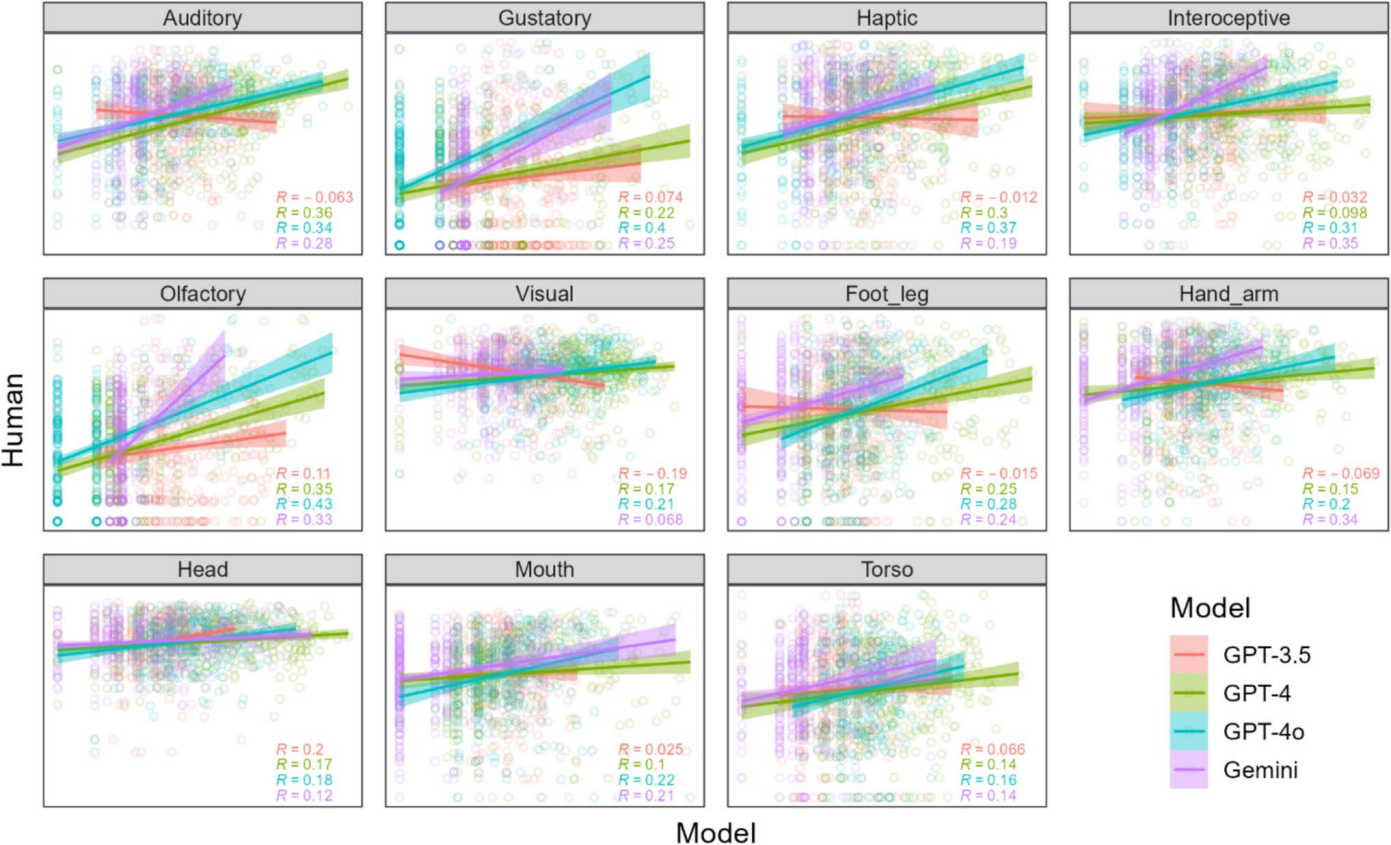
Scatter plots of LLM rating SDs (*X*-axis) against human rating SDs (*Y*-axis) for each sensorimotor variable in Experiment 2, with 384 points per plot corresponding to the respective human and LLM rating SDs for 384 perception or action words. Multiple correlation coefficients R for each model are shown in the bottom *right corner* of each plot. *X*- and *Y*-axis ranges are the same within each plot, and set to minima and maxima of each variable, so a perfect positive relationship falls along the diagonal

Correspondences between human and LLM rating SDs are shown in Fig. 4. Correlation coefficients were highly reliable, albeit only weakly positive overall, as tested using a linear mixed-effects model with LLM rating SDs (*Z*-scored) as a predictor of human rating SDs, plus a random effect of word: *b* = 0.16, *p* < 2e-16. And once again, LLM model was added as a fixed effect to confirm a weaker correspondence for GPT-3.5 compared with GPT-4 (*b* = 0.14, *p* = 2.21e-14), GPT-4o (*b* = 0.21, *p* < 2e-16) and Gemini (*b* = 0.18, *p* < 2e-16). Like rating means, correlations were stronger overall for perceptual versus action variables (*b* = 0.06, *p* = 2.05e-05), but correlations were generally weak and there were no apparent patterns within action or perception variables. As a result, there was no reliable correspondence between the SD of rating means and SD correlations across variables, *r* = 0.05.

#### Original versus binned word lists

Next, we tested whether correspondences between human and LLM ratings for Vision and Mouth variables were affected by binning word lists according to these variables and using their exemplars as control and calibration words. Correlation coefficients for rating means and SDs are shown in Table 1 for the two targeted variables in the original and binned conditions, for each of the four models tested. For rating means, correlation coefficients for the Vision and Mouth variables were higher for the binned word lists for all four models, ranging from a difference of 0.10 to 0.32. By contrast, there was no consistent pattern for rating SDs, with some correlations dropping into negative territory in the binned conditions, and others rising substantially across different models and the two variables.

**Table 1:** Correlation coefficients in Experiment 2 as a function of sensorimotor variable (ACTION or PERCEPTION), context (Original or Binned), and model. Averages shown in bold

	ACTION			PERCEPTION		
*Means*	*Original*	*Binned*	*Diff*	*Original*	*Binned*	*Diff*
*GPT-3.5*	0.16	0.27	0.11	0.03	0.32	0.28
*GPT-4*	0.58	0.75	0.18	0.64	0.75	0.11
*GPT-4o*	0.66	0.75	0.10	0.38	0.70	0.32
*Gem-1.5*	0.45	0.57	0.12	0.35	0.67	0.32
**AVG**	**0.46**	**0.59**	**0.13**	**0.35**	**0.61**	**0.26**
*SDs*	*Original*	*Binned*	*Diff*	*Original*	*Binned*	*Diff*
*GPT-3.5*	0.03	– 0.37	– 0.40	– 0.19	– 0.25	– 0.06
*GPT-4*	0.10	0.22	0.11	0.16	0.32	0.16
*GPT-4o*	0.22	0.53	0.31	0.21	0.04	– 0.16
*Gem-1.5*	0.21	0.31	0.10	0.07	– 0.17	– 0.24
*AVG*	**0.14**	**0.17**	**0.03**	**0.06**	**– 0.01**	**– 0.08**

To test the reliability of the binning effect on rating means and rating SDs, we compared linear effects models including only data for the binned variables. Linear regression models with LLM rating means (*Z*-scored) as a predictor of human rating means and binning as a fixed effect confirmed that correlation coefficients were more reliable for the binned condition compared to the original condition for both Vision (*b* = 0.26, *p* = 6.09e-16) and Mouth (*b* = 0.13, *p* = 3.93e-05). We repeated these tests using rating SDs, which showed no difference for Mouth (*b* = 0.03, *p* = 0.386), and a marginally reliable decrease in correlation strengths for Vision in the binned condition (*b* = – 0.08, *p* = 0.031). This difference was carried entirely by GPT-3.5, whereas more advanced models trended in the opposite direction, consistent with the effect of binning for rating means.

### Experiment 2 summary

Results from Experiment 2 further challenged the recall account of correlations between human and LLM ratings. Ratings were again reliably correlated with respect to both their means and SDs, and differences in correlations for rating means across variables were partly explained by differences in ambiguity. Moreover, the contexts of word lists had a pronounced effect on the correspondence between human and LLM rating means: Correlation coefficients increased for the Mouth and Vision variables when lists were binned and calibrated according to them. There is no apparent reason for surrounding words to affect how well means and SDs can be recalled from training data. If LLMs averaged individual ratings from word lists, one would expect *stronger* correlations for the original lists, which should serve as cues to recall. By contrast, the observed effect is expected if the surrounding words calibrate the generalization of word usage in training data. The anchoring of exemplars for sensorimotor variables used to sort word lists may better guide the assembly of rating functions based on human word usage.

While LLM ratings were correlated with human ratings, as in Experiment 1, correlations were not as strong in Experiment 2. Different words were used, and multiple variables were prompted simultaneously, so it is not clear whether weaker correlations were due to extraneous factors or differences in the variables between experiments. We checked whether it might be because words are generally more concrete in Experiment 2, which may lower correlations (Trott, 2024), but words were actually less concrete on average compared with Experiment 1 (3.09 versus 3.72 using norms from Brysbaert et al., 2014). Instead, one might expect LLMs to be less human-like in Experiment 2 because LLMs have no bodily experience to generalize from for sensorimotor ratings (Conde et al., 2025; Xu et al., 2025). We further test the recall account and the limits of LLM generalization in Experiment 3.

## Experiment 3: Speeded response tasks

The generalization account posits that LLMs use context to guide the assembly of generalized word rating functions based on patterns of word usage in training data. On this account, both humans and LLMs assembled similar rating functions in Experiment 1 because psycholinguistic variables are based on patterns of human word usage that are manifest in training data. The same interpretation holds for Experiment 2, except only humans can draw upon bodily experiences for sensorimotor ratings, and these experiences may not be fully encoded in patterns of word usage. This difference might explain the lower human-LLM correlations in Experiment 2, but the evidence is muddied by confounding factors.

Experiment 3 was designed to provide a further test of the recall account and the hypothesized limits of LLM generalization, which can only derive from patterns available in training data. Word ratings are *offline* measures because they do not tap into the moment-by-moment unfolding of lexical processes in real time. By contrast, there is a very large literature on studies of *online* measures of lexical processing, like response times in lexical decision and word naming tasks (Katz et al., 2012). The underlying lexical processes unfold on timescales of milliseconds that are categorically faster than the timescales of word usage captured in LLM training data.

The generalization account predicts that assembled LLM response time functions should be less human-like than LLM word ratings because LLM training data do not directly reflect the millisecond timescales of lexical processing. By contrast, the recall hypothesis makes no such prediction. Response time data have been reported in psycholinguistic studies of words for decades, including the publication of large corpora like the norming studies from Experiments 1 and 2. Therefore, if human-LLM correlations are due to LLM recall of published human data, then correlations should be at least as strong for response times as for word ratings. One might even expect stronger correlations on the recall account because LLM training data should include the far larger and richer literature on lexical processing, including greater availability of multiple latencies per word that may drive LLM responses and their variability.

### Methods

The 390 words selected for Experiment 1 were also selected to have word naming data in the SCOPE metabase from the English Lexicon Project (Balota et al., 2002). We downloaded naming latency means and intra-word SDs, along with four factors known to account for variance in latencies: Word length in number of letters, word frequency (log transformed) as measured by the number of different movies it appears in (Brysbaert & New, 2009), spelling-to-sound regularity as measured by feed-forward onset consistency (Chee et al., 2020), and initial phoneme as a measure of onset energy that takes into account the time needed for acoustic energy to cross threshold once articulation has begun (Kawamoto et al., 1998; Rastle & Davis, 2002). Onset energy was coded in terms of initial phonemes that were stops, affricates, and fricatives as a value of 1, with all other phonemes coded as 0, to denote the extra time needed for energy to ramp up compared with other phonemes.

We cannot measure naming latencies directly from LLMs because their measurable time course of processing does not vary with the content of prompts and responses (longer prompts and responses may take longer to process, but in our study, average token length was not varied across conditions). However, we can prompt LLMs with the word naming task and request average naming latencies for a list of words, analogous to Experiments 1 and 2 (see Supplementary Materials for prompts).

### Results

**Fig. 5:**
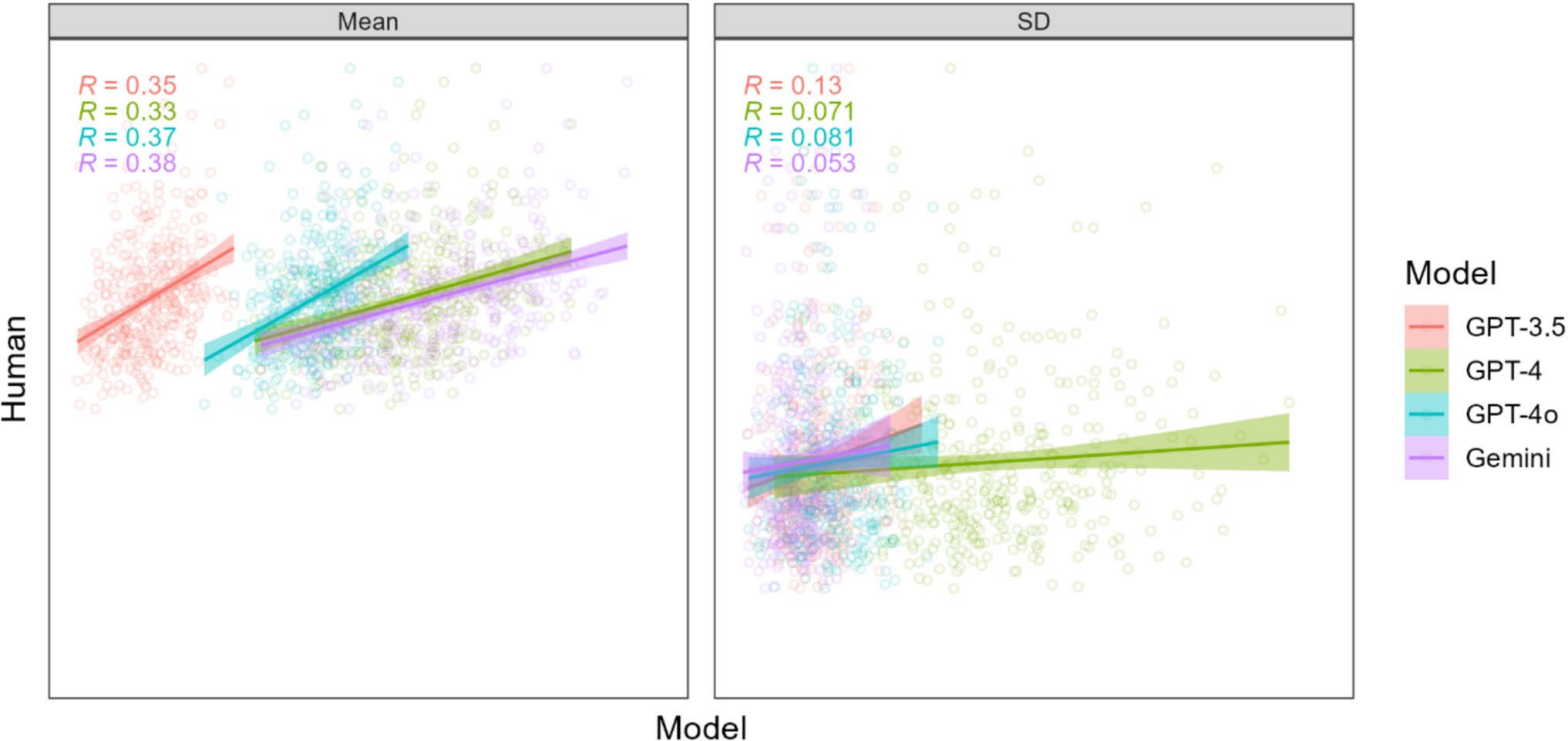
Scatter plots of LLM latency means and SDs (*X*-axes) against human latency means and SDs (*Y*-axes) for word naming for the 390 words used in Experiment 3. Multiple correlation coefficients, *R,* for each model are shown in each plot. *X-* and *Y*-axis ranges are the same within each plot, and set to minima and maxima of each variable, so a perfect positive relationship falls along the diagonal.

Figure 5 shows scatter plots for human versus LLM naming latencies, both means and SDs. The plots and *R* values show that correlation coefficients for latency means are noticeably weaker than those for rating means in Experiments 1 and 2. A linear mixed-effects model with LLM means as a predictor for human means, plus a random effect of word, showed a reliable relationship overall (*b* = 0.36, *p* < 2e-16). There were also reliable but very weakly positive relationships for latency SDs (*b* = 0.09, *p* = 0.000767).

Results so far are consistent with the generalization account, but we must consider two differences between latencies and word ratings. First, it is possible that correlations are lower for latencies because there is a lower limit on how much of their variance can be explained, compared with word ratings. To provide a baseline level of correlation, we used the four factors mentioned earlier in a linear regression model with mean naming latencies for humans as the dependent measure. The predictors accounted for a total of 34% of the variance (details below), whereas the LLMs accounted for only 10–15% of the same variance. Therefore, lower correlations in Experiment 3 were not due to limited explainability.

The second difference to consider is that LLMs may be poor at estimating relatively large numbers representing quantities like hundreds of milliseconds for latencies, whereas LLMs might generalize better for measures like word ratings in the single digits. We tested the LLM's ability to estimate large numbers for a different but related measure, i.e., word frequency, using the Brysbaert and New (2009) movie subtitle method. Ten frequency counts per word were logged and then averaged, and for the advanced models, correlations with human naming latencies were about the same as for the original word frequency counts, from about – 0.38 to – 0.43 (also see Brysbaert et al., 2024). We can conclude that LLMs were able to generate accurate estimates of word frequencies over several orders of magnitude, which means that their shortcomings as models of naming latencies are not due to the scale of the dependent measure.

It appears that LLMs assembled generalized latency functions that were not well-matched to the millisecond timescales of lexical processing. We can further investigate this mismatch by examining the latency functions in more detail to uncover similarities and differences between human and LLM lexical processes. Our linear regression model from above provides an empirical measure of the mean latency function for humans, in that standardized betas quantify the contributions of individual predictors to the fitted regression. We can fit the same regression model to LLM naming latencies and use the fitted parameters to compare the functions assembled by LLMs for word naming with those enacted in real time by humans.

Regression model parameters and statistics are shown in Table 2 for the means of human latencies as well as the four models. For human latencies, all four predictors made moderate and highly reliable contributions to the regression model. By comparison, model latency functions generally gave too much weight to word length and nothing to onset energy. A mixed-effects linear regression model with word length as a predictor of latency and a random effect of word confirmed that the effect of length on latencies was significantly stronger for each LLM model compared to the human data: *b* = 0.29, *p* = 4.62e-14 for GPT-3.5; *b* = 0.15, *p* = 0.000109 for GPT-4; *b* = 0.21, *p* = 4.37e-08 for GPT-4o; *b* = 0.37, *p* < 2e-16. The same model with onset energy as a predictor instead of word length also confirmed that the effect of onset energy on latencies was significantly weaker for each LLM model compared to the human data: *b* = −0.56, *p* = 3.51e-07 for GPT-3.5; *b* = – 0.67, *p* = 1.47e-09 for GPT-4; *b* = – 0.57, *p* = 2.65e-07 for GPT-4o; *b* = – 0.62, *p* = 1.34e-08. No significant differences were found when spelling-to-sound consistency and word frequency were used as predictors.

**Table 2:** Experiment 3 regression model parameters and statistics for the four predictors—word frequency and length (Freq and Length), feed-forward onset consistency (FF cons), and onset energy (Onset). Highly reliable *p* values shown in bold, moderately reliable in italics

	HUMAN	GPT-3.5	GPT-4	GPT-4o	Gem-1.5
	Standardized beta				
Freq	– 0.37	– 0.22	– 0.22	– 0.35	– 0.23
Length	0.23	0.67	0.47	0.53	0.75
FF cons	– 0.18	– 0.03	– 0.10	– 0.04	– 0.07
Onset	0.26	– 0.01	– 0.06	0.00	– 0.04
	*t*-statistic				
Freq	– 8.72	– 6.22	– 5.22	– 9.34	– 8.24
Length	5.36	19.04	11.06	14.19	26.44
FF cons	– 4.36	– 0.98	– 2.34	– 1.14	– 2.62
Onset	6.20	– 0.31	– 1.37	– 0.05	– 1.52
	*p* value				
Freq	**8e-17**	**1e-09**	**3e-07**	**8e-19**	**3e-15**
Length	**1e-07**	**2e-57**	**7e-25**	**5e-37**	**1e-88**
FF cons	**2e-05**	0.329	*0.020*	0.253	*0.009*
Onset	**1e-09**	0.758	0.173	0.958	0.131

### Experiment 3 summary

The results of Experiment 3 provided further evidence against the recall account and for generalization. The recall account predicts equal or stronger correlations for naming latencies compared with word ratings, but the opposite result was found. Alternate explanations were ruled out, and latency functions were further examined and compared with respect to four well-known predictors of word naming. Results showed how LLMs latency functions deviated from the human data in ways explained by generalization.

Specifically, LLMs failed to account for onset energy, which is a physical, temporally fine-grained effect based on the relationship between speech articulation and acoustics. The effect unfolds on a timescale not captured in patterns of word co-occurrences. LLMs also failed in putting too much weight on word length, whose correlations with latencies were 2–3 times larger for LLMs compared with humans. It appears that LLMs incorrectly generalized the association of word length with reading difficulty, whereas skilled readers can process letters and sounds in parallel during speeded naming tasks (Hudson & Bergman, 1985; Kawamoto et al., 1999). As with onset energy, the timescale of online lexical processing is faster than patterns of word co-occurrences, and hence opaque to LLM assembly of latency functions.

## General discussion

We set out to investigate two competing hypotheses about how LLMs generate lexical functions such as psycholinguistic ratings and naming latencies. If LLMs assemble generalized lexical functions, they might serve as useful models of lexical processing beyond the methodological role of simulating human data. But if LLMs recall published measurements from training data, they would be ill-suited as models of lexical processing.

Results favored generalization over recall. Psycholinguistic ratings generated by LLMs were highly correlated with mean human ratings for the advanced models, with nearly half of the correlation coefficients > 0.9 for GPT-4o. Intra-word variability in LLM ratings was also correlated with human variability in terms of intra-word SDs. For both means and SDs, differences in strengths of correlations across variables were partly explained by differences in their ambiguity, which is expected by generalization. A recall function might explain correlations overall if individual ratings found in training data are distributed according to the published means and SDs, but the Glasgow norming study only published the means and SDs themselves. Moreover, such data contamination does not readily explain the effect of ambiguity on correlations.

Experiment 2 provided further evidence against recall and for generalization. Sorted word lists were composed to calibrate with human ratings by providing LLMs with exemplar words for the sensorimotor variables that were sorted. Calibration can assist with a generalized rating function, but not a recall function. The latter should instead be aided by having words appear in their originally published contexts as cues to recall. Correlations were stronger for LLM ratings in the sorted lists, consistent with the generalization account.

Correlations should also be stronger when more lexical data are available to recall and average, but we found the opposite in Experiment 3. Word naming is a well-studied task with decades of published data, including several large-scale corpora. If psycholinguistic ratings are included in LLM training data, then it must also be the case that the large number of published naming latencies are also included. Yet, LLM latencies were less correlated with human data compared with ratings in Experiments 1 and 2, and differences between the two measures were ruled out as possible explanations. Moreover, LLM latencies deviated from human latencies in functional ways: LLMs were more strongly influenced by word length and uninfluenced by the acoustic properties of word onsets. We see no way for data contamination to explain these deviations.

Generalization, by contrast, provides a natural explanation for the functional deviations of LLMs and the other results that challenge the recall account. LLMs are hypothesized to use training data and context to guide the assembly of lexical functions when prompted. The assembly of latency functions is based on the slower timescales of patterns in word co-occurrences that comprise their training data. Faster timescales that affect human latencies, such as millisecond dynamics of reading and speech production, are not encoded in LLM training data and so did not factor into the assembled functions.

More generally, human lexical processing is inherently contextual in terms of semantics, phonology, and other factors (Levy et al., 2021). LLM responses are also inherently contextual, and the effects of context on language processing are human-like in some respects (Cai et al., 2023). Our study adds psycholinguistic and sensorimotor ratings of words to this growing body of evidence. Correlations with sensorimotor ratings were not as strong as psycholinguistic ratings, which suggests that textual training data may not encode human sensorimotor experience as well as psycholinguistic variables. These results replicate recent studies (Conde et al., 2025; Xu et al., 2025) that also prompted LLMs for ratings from the Glasgow and Lancaster norming studies and found weaker correlations for sensorimotor ratings. Additional analyses comparing patterns of rating similarities across words and variables suggested closer conceptual alignment (Rane et al., 2024) between humans and LLMs for psycholinguistic variables.

### LLMs as models of lexical processing

Our results show that LLMs can serve as accurate models of offline lexical processing when context can guide the assembly of lexical functions based on generalizations from patterns in training data. As such, LLMs may be used to generate psycholinguistic data, but the composition of all stimuli presented, as well as the larger context of the experimental protocol and conditions, should be carefully considered. Correlations show that generated data can be more representative of human behavior when averaged over an appropriate sampling of contexts.

We can also consider whether the use of context to guide the assembly of lexical functions informs us about how human language users process words in their language. Most directly, human lexical processes may also be assembled in context, rather than pre-compiled during language learning. This implication calls into question theories of the mental lexicon that are architecturally fixed. For example, debates about models of word reading traditionally centered around system components that were built or trained to perform specific functions like mapping letters to sounds or activating pre-determined representations (Coltheart, 2006; Joanisse & McClelland, 2015). By contrast, LLMs are not built to generate word ratings or naming latencies or any psycholinguistic data, and neither are humans built to perform tasks contrived for experiments (Van Orden et al., 2001).

Instead, LLMs show us how performance in psycholinguistic tasks can emerge from more basic processes of language learning, such as using prediction to learn a generative model of language. LLMs also show us how context is essential to guiding the assembly of lexical functions, just as the results of psycholinguistic experiments can hinge on details in the experimental protocols and stimulus sets. In both LLMs and humans, the ability to perform psycholinguistic tasks is latent in language learning and only takes shape on demand (Elman, 2009; also see Kello & Van Orden, 2009).

LLMs hold the promise of a new approach to modeling lexical processes, but there are several challenges to address moving forward. First, while our results provide evidence against data contamination as a significant factor, it is possible that published data played some as yet undiscovered role in LLM responses. Other studies have found that some contexts will guide LLMs to assemble recall functions for some elements of their training data (Bender et al., 2021; Carlini et al., 2021). Work is ongoing to develop a theoretical understanding of data contamination, mainly driven by privacy and security concerns, and this work is needed so that researchers can know up front whether a given prompt will draw published data into an LLM response.

Work on data contamination is one example of broader efforts to develop theories of LLMs, along the lines of studies on mechanistic interpretability (Conmy et al., 2023). LLMs are still black box models despite decades of work on theories of artificial neural networks (Samek et al., 2017). Their nonlinearities, dependencies on training data and parameters, and interactions with prompts all pose deep challenges to using LLMs as models of human behavior or any other phenomena. In our case, we lack a detailed theory of contextual assembly in LLMs. Limits in understanding might limit their usefulness as models of human language, but they are already valuable as comparative models, and progress is being made on LLM theories (Zhao et al., 2024) and methods of analysis (Rai et al., 2024).

Transformers are amenable to analysis but only if code and data are made available, and only if researchers have the computing resources needed to train models. Results across the different models tested herein showed that the contextual assembly of lexical functions was most robust in the largest proprietary models. Therefore, for now at least, experimental studies like the current one are needed to study robust assembly of lexical functions in LLMs. Most researchers may not have access to the data and computing resources needed to train LLMs that are sufficiently human-like for modeling purposes. Fortunately, work is underway to make LLMs more efficient in terms of performance relative to the amount of data and computing power (Ding et al., 2023).

Finally, LLMs may need to become more multimodal in their training data and interactions with the external world. Online lexical processes are situated in timescales of milliseconds to seconds, so current LLMs need to generalize from timescales of patterns in textual training data. Results herein indicate that such generalization across timescales may hinder the assembly of human-like naming latency functions and other fast online processes. It is encouraging that studies have found relationships between LLM representations and intracranial electroencephalogram recordings (Mischler et al., 2024) as well as hemodynamic activity (Kumar et al., 2024) during language processing. These studies show a capacity for fine-grained relationships between humans and LLMs that might expand with training data that encompass the fine-grained multimodal dynamics of language interactions (Vong et al., 2024).

The data and materials for all experiments are available at https://osf.io/crqy2/?view_only=94c879d81a544efc902a7869621409de and none of the experiments were preregistered.

## Supplementary Information

Below is the link to the electronic supplementary material.

Supplementary file1 (PDF 189 KB)

## Data Availability

The datasets generated and analyzed during the current study are available at https://osf.io/crqy2/?view_only=94c879d81a544efc902a7869621409de

## Code availability

The code used to generate and analyze the datasets is also in the repository above.
