# Supplementary material for: Contextual assembly of lexical functions in large language models
Source: Behav Res Methods. 2025 Dec 8;58(1):19. doi: 10.3758/s13428-025-02898-7 (PMC12686107; doi:10.3758/s13428-025-02898-7)
Supplement: Supplementary file 1 — Supplementary file1 (PDF 189 KB) [file 13428_2025_2898_MOESM1_ESM.pdf]

Appendix

Experiment 1 Prompts

[*System*] The user will describe a feature of words and how to quantify it and assign values to words. The user will then provide a list of words. Respond only with the list of words and their corresponding values, one 'word,value' pair per line.

[*Age of Acquisition*] A word's age of acquisition is the age at which the word is initially learned in spoken or written form, whichever comes first. Rate the average age of acquisition for each of the following words on a continuous linear scale from 1.0 (acquired before two years old) to 7.0 (acquired after 12 years old):

[*Concreteness*] Concreteness is a measure of how concrete or abstract something is. A word is ABSTRACT if it represents a concept or idea. In contrast, a word is CONCRETE if it represents something that exists in a definite physical form in the real world. Rate how concrete each of the following words is on a continuous scale from 1.0 (VERY ABSTRACT) to 7.0 (VERY CONCRETE), with the midpoint being neither especially abstract nor concrete:

[*Arousal*] Arousal is a measure of calmness versus excitement. A word is UNAROUSING if it makes one feel relaxed, calm, sluggish, dull, or sleepy. A word is AROUSING if it makes one feel stimulated, excited, frenzied, jittery, or wide-awake. Rate how arousing each of the following words is on a continuous scale of 1.0 (VERY UNAROUSING) to 9.0 (VERY AROUSING), with the midpoint representing moderate arousal:

[*Dominance*] Dominance is a measure of the degree of control felt by a person. A word can make a person feel DOMINANT, influential, in control, important, or autonomous. In contrast, a word can make a person feel CONTROLLED, influenced, cared-for, submissive, or guided. Indicate how each word makes a person feel on a continuous scale of 1.0 (VERY CONTROLLED) to 9.0 (VERY DOMINANT), with the midpoint being neither controlled nor dominant:

[*Familiarity*] Familiarity is a measure of how familiar something is. A word is very UNFAMILIAR if you rarely see/hear it and it is relatively unrecognizable. In contrast, a word is very FAMILIAR if you see/hear it often and it is easily recognizable. Rate how familiar each of the following words is on a continuous scale of 1.0 (VERY UNFAMILIAR) to 7.0 (VERY FAMILIAR), with the midpoint representing moderate familiarity:

[*Gender*] Gender is a measure of the association of a word with attributes or behaviors that are either female or male in nature. The gender of a word is FEMININE if it is linked to female attributes or behaviors. In contrast, the gender of a word is MASCULINE if it is linked to male attributes or behaviors. Rate the gender of each of the following words on a continuous scale

of 1.0 (VERY FEMININE) to 7.0 (VERY MASCULINE), with the midpoint being neuter (neither feminine nor masculine):

[*Humorousness*] Humorousness is a measure of how humorous something is. A word is HUMORLESS if it is dull or unfunny. A word is HUMOROUS if it is absurd, amusing, hilarious, playful, silly, whimsical, or laughable. Rate the humorousness for each of the following words on a continuous scale of 1.0 (HUMORLESS) to 5.0 (HUMOROUS), with the midpoint being neutral:

[*Imageability*] Imageability is a measure of how easy or difficult something is to imagine. A word is UNIMAGEABLE if it represents something that is very difficult to imagine or picture. In contrast, a word is IMAGEABLE if it represents something that is very easy to imagine or picture. Rate how imageable each of the following words is on a continuous scale of 1.0 (VERY UNIMAGEABLE) to 7.0 (VERY IMAGEABLE), with the midpoint being moderately imageable:

[*Semantic Size*] Size is a measure of dimension, magnitude, or extent. A word represents something SMALL if it refers to things or concepts that are little. In contrast, a word represents something BIG if it refers to things or concepts that are large. Rate the semantic size for each of the following words on a continuous scale of 1.0 (VERY SMALL) to 7.0 (VERY BIG), with the midpoint being neither small nor big:

[*Socialness*] Words differ in the degree to which they can be considered social. Some words clearly refer to social things, social qualities or to social actions or events (e.g., 'trustworthy') whereas, for other words, the relationship to society or social interactions might only become apparent after a period of intense thought (e.g., 'promotion'), or not at all (e.g., 'chair'). A word referring to something that has clear social relevance should be given a high socialness rating (at the upper end of the numerical scale). A word referring to something lacking in social relevance should be given a low socialness rating (at the lower end of the scale). A word referring to something that is not fundamentally social but has some social elements (e.g., 'smartphone'), or can be thought of as social in some circumstances (e.g., 'event'), should be given an intermediate socialness rating. It is important to base ratings on the degree of the social relevance of the word's meaning and not whether the meaning is prosocial versus antisocial or evokes positive/negative associations. For example, the word 'fight' should be given a high socialness rating because it refers to a type of interaction between people, even though the interaction is antisocial. Rate the socialness of each of the following words on a continuous scale from 1.0 (low socialness) to 7.0 (high socialness):

[*Valence*] Valence is a measure of value or worth. A word is NEGATIVE if it represents something considered bad, whereas a word is POSITIVE if it represents something considered

good. Rate the valence of each of the following words on a continuous scale from 1.0 (VERY NEGATIVE) to 9.0 (VERY POSITIVE), with the midpoint representing NEUTRAL:

Experiment 1 Other Model Results

We collected data for the psycholinguistic variables and word lists with four additional models, three of which were state-of-the-art proprietary models: gpt-4.5-preview-2025-02-27, gemini-2.0-flash, and claude-3-7-sonnet-20250219. Correlations with human rating means and SDs are shown in the table below. Coefficients were about the same for GPT 4.5 versus 4o (see Figure 1 of main text), slightly stronger for Gemini 2.0 versus 1.5, and correlations with rating means were strongest overall for Claude 3.7. However, these differences were small, and the pattern of results was the same as for the models reported in the main text.

We also included one open-source model from a provider that was rated highly according to the Hugging Face leaderboard at the time: Qwen3-4B-Instruct-2507. Ratings from the open-source model were substantially less correlated than for the proprietary models, and appeared to fail for the gender variable and some of the SD correlations.

	**GPT 4.5**		**Gemini 2.0**		**Claude 3.7**		**Qwen3-4B**	
**Variable**	*Means*	*SDs*	*Means*	*SDs*	*Means*	*SDs*	*Means*	*SDs*
AoA	0.93	0.27	0.88	0.27	0.94	0.23	0.69	0.03
Arousal	0.64	-0.04	0.53	-0.04	0.72	0.11	0.68	0.30
Concreteness	0.94	0.38	0.93	0.37	0.96	0.45	0.84	-0.15
Dominance	0.7	0.39	0.6	0.4	0.64	0.31	0.60	0.26
Familiarity	0.82	0.6	0.77	0.57	0.83	0.51	0.75	-0.17
Gender	0.9	0.32	0.86	0.08	0.9	0.13	0.19	0.06
Humor	0.67	0.19	0.64	0.37	0.7	0.11	0.46	0.14
Imageability	0.93	0.37	0.91	0.62	0.95	0.6	0.56	-0.26
Semantic Size	0.79	0.12	0.88	0.16	0.88	0.15	0.77	0.15
Socialness	0.92	0.51	0.9	0.32	0.92	0.39	0.81	0.24
Valence	0.96	0.27	0.94	0.21	0.97	0.39	0.88	0.29
**AVERAGE**	**0.84**	**0.31**	**0.8**	**0.3**	**0.85**	**0.31**	**0.66**	**0.08**

Experiment 1: Correlation Coefficients for Psycholinguistic Means and SDs

Means	GPT-3.5	GPT-4	GPT-4o	Gemini
AoA Rating	0.71	0.90	0.92	0.85
Arousal	0.67	0.56	0.60	0.61
Concreteness	0.83	0.95	0.94	0.91
Dominance	0.67	0.60	0.70	0.60
Familiarity	0.66	0.79	0.82	0.80
Gender	0.75	0.89	0.87	0.87
Humorousness	0.47	0.67	0.64	0.60
Imageability	0.25	0.92	0.91	0.87
Semantic Size	0.69	0.87	0.78	0.76
Socialness	0.79	0.90	0.91	0.89
Valence	0.93	0.95	0.96	0.93

SDs	GPT-3.5	GPT-4	GPT-4o	Gemini
AoA Rating	0.04	0.22	0.25	0.04
Arousal	0.38	0.02	0.13	0.04
Concreteness	0.40	0.58	0.42	0.14
Dominance	0.27	0.28	0.39	0.27
Familiarity	0.33	0.48	0.39	0.43
Gender	0.02	0.10	0.20	0.20
Humorousness	0.05	0.32	0.24	0.09
Imageability	0.09	0.54	0.47	0.37
Semantic Size	0.07	0.23	0.09	0.16
Socialness	0.08	0.38	0.45	0.47
Valence	0.22	0.26	0.39	0.17

Experiment 2: Correlation Coefficients for Sensorimotor Means and SDs

Means	GPT-3.5	GPT-4	GPT-4o	Gemini
Auditory	0.18	0.65	0.61	0.55
Gustatory	0.32	0.89	0.93	0.85
Haptic	0.21	0.76	0.73	0.66
Interoceptive	0.22	0.57	0.64	0.61
Olfactory	0.30	0.83	0.84	0.77
Visual	0.03	0.65	0.38	0.35
Foot/Leg	0.20	0.63	0.63	0.55
Hand/Arm	0.16	0.70	0.66	0.65
Head	0.27	0.54	0.63	0.49
Mouth	0.16	0.58	0.66	0.45
Torso	0.19	0.62	0.63	0.47

SDs	GPT-3.5	GPT-4	GPT-4o	Gemini
Auditory	-0.06	0.36	0.34	0.28
Gustatory	0.07	0.22	0.40	0.25
Haptic	-0.01	0.30	0.37	0.19
Interoceptive	0.03	0.10	0.31	0.35
Olfactory	0.11	0.35	0.43	0.33
Visual	-0.19	0.17	0.21	0.07
Foot/Leg	-0.02	0.25	0.28	0.24
Hand/Arm	-0.07	0.15	0.20	0.34
Head	0.20	0.17	0.18	0.12
Mouth	0.03	0.10	0.22	0.21
Torso	0.07	0.14	0.16	0.14

Experiment 2 Prompts

[*System, 5 or 6 ratings for Action or Perception*] Encode the task and then respond with each word or phrase along with its five ratings, separated by commas, using the following template for each line: word/phrase,rating,rating,rating,rating,rating

[*Action, example order of body parts*] On a scale of 0 (not at all experienced) to 5 (greatly experienced), rate the extent to which someone might experience an action with each of five body parts (foot/leg,hand/arm,mouth/throat,head excluding mouth,torso) for each of the following 58 words and phrases, one word or phrase per line, 58 lines in all:

[*Perception, example order of body parts*] On a scale of 0 (not experienced at all) to 5 (greatly experienced), rate the extent to which someone might use each of six perceptual modalities (feeling through touch,hearing,sensations inside the body,smelling,seeing,tasting) to experience each of the following 58 words and phrases, one word or phrase per line, 58 lines in all:

Experiment 3 Prompts

[*System*] The user will describe a psycholinguistic task followed by a list of words. Respond only with the list of words and their corresponding latency values in milliseconds, one 'word,value' pair per line.

[*Naming*] Imagine that words appear one at a time on a computer screen, and the task is to read each word out loud as quickly and accurately as possible. For each of the following words, report the average response latency as measured by the time in milliseconds from when the word appears to the onset of acoustic energy:

[*Frequency*] The frequency of a word is based on its number of occurrences in a corpus of text. Imagine a large number of films with American English subtitles comprising a corpus of one million word occurrences. Estimate the number of occurrences in the corpus for each of the following words:
